# Functional Response and Control Potential of *Orius sauteri* (Hemiptera: Anthocoridae) on Tea Thrips (*Dendrothrips minowai* Priesner)

**DOI:** 10.3390/insects12121132

**Published:** 2021-12-17

**Authors:** Qiuping Zhang, Ruifang Zhang, Qiuqiu Zhang, Dezhong Ji, Xia Zhou, Linhong Jin

**Affiliations:** State Key Laboratory Breeding Base of Green Pesticide and Agricultural Bioengineering, Key Laboratory of Green Pesticide and Agricultural Bioengineering, Ministry of Education, Guizhou University, Huaxi District, Guiyang 550025, China; gs.zhangqp16@gzu.edu.cn (Q.Z.); gs.rfzhang16@gzu.edu.cn (R.Z.); gs.zhangqq18@gzu.edu.cn (Q.Z.); gs.dzji19@gzu.edu.cn (D.J.)

**Keywords:** *O. sauteri*, tea thrips, natural enemy, functional response, predation

## Abstract

**Simple Summary:**

Globally, the tea plant is an economically significant beverage crop, especially in China. Tea thrips are one of the most destructive pest species in the tea industry. *Orius sauteri,* a predatory insect, has been introduced as a natural enemy for other pests in different plants. In this paper, we primarily evaluate the functional response and control potential of *Orius sauteri* on tea thrips for the purpose of applying *Orius sauteri* as their natural enemy. We studied the functional response and searching rate of *O. sauteri*, and the results indicated that *O. sauteri* possesses good control potential regarding the thrips.

**Abstract:**

This study aimed to clarify the functional response and control potential of *O*. *sauteri* in relation to tea thrips. The functional response, interference response, and control potential of *O. sauteri* on adult tea thrips, in different insect stages and environment temperatures, were studied. The results showed that the predation of *O. sauteri* against tea thrips was positively correlated with prey density, while the effects of searching for *O. sauteri* on the adult tea thrips were negatively correlated with prey density. The predation effects of *O. sauteri* on tea thrips were also influenced by prey density, which indicated that there was an intra-specific interference response from predators to tea thrips. The population density of tea thrips was significantly decreased, and *O. sauteri* showed a remarkable ability to control them when the benefit-to-harm ratio was 3:100.

## 1. Introduction

The tea plant *Camellia sinensis* (L.) O. Kuntze is an economically significant beverage crop, especially in China [1]. Tea thrips (*Dendrothrips minowai* Priesner) are minute, slender insects with fringed wings that feed on tea leaf sap by puncturing leaves and sucking up the contents; this affects the growth and development of the tea tree. Tea thrips have several generational overlaps [2] in a year and can cause a decline in tea quality, yield, and, consequently, the income of tea farmers. At present, the application of pesticide is the main method for controlling pest insects, but there are concerns regarding pesticide residue and its adverse effects on the natural enemies of tea pests [3]. The application of sustainable pest management practices would reduce the over-reliance on pesticides [4]. Biological control plays a substantial role in the comprehensive prevention and control of integrated pest management in green agriculture. It is an economically and environmentally friendly approach for controlling the production of pests. Natural enemy-mediated control can also be helpful in tea gardens [5]. At the same time, increasing tea consumption requires improved standards for the control of tea pests. As such, selecting and utilizing natural enemies are of great significance in the biological control of tea pests.

*O*. *sauteri* (Poppius) (*Heteroptera: Anthocoridae*) is a polyphagous predator and plays an important role in controlling pests in forests, orchards, greenhouses, and farmland [6,7,8]. It is often used as a natural enemy and can consume many varieties of prey [9,10,11]. *O. sauteri* is registered as a biocontrol agent and its predation capability regarding thrips in eggplant has been evaluated [6]. However, *O*. *sauteri* has rarely been reported to possess a biological control effect on tea thrips, including its response to plant and prey availability [12,13].

To evaluate the control effect of *O*. *sauteri* as a natural enemy to tea thrips, this study examined the functional response, searching ability, and selective predation of *O. sauteri* (2nd and 4th nymphs and adults) on adult tea thrips. The present results might provide a reliable basis for a proper evaluation of the biological control potential of *O. sauteri* on tea thrips in tea gardens.

## 2. Materials and Methods 

### 2.1. Insect Collection 

Tea thrips were collected from the pesticide-free tea garden in Qingyan (26.35 degrees north and 106.42 degrees east, Guiyang, Guizhou province, China). The collected insects and tea trees were kept and raised in a laboratory with artificial climate incubators at 25 ± 1 °C, 75 ± 5% relative humidity (RH) 16 h:8 h light (L)/dark (D).

*O. sauteri* was purchased from Beijing Kuoye Tianyuan Biotechnology Co., Ltd. (Beijing, China) as described previously [13]. In addition, the feeding method of the indoor population was established, and insects were starved for 24 h before starting the assay. Both nymphs and adults were fed with rice meal moth eggs (*Corcyra cephalonica*) and reared in separate containers in a climate-controlled room (25 ± 1 °C, 70 ± 5% RH, 16:8 L/D) at Guizhou University. Additionally, frozen–fresh rice meal moth eggs were provided by colleagues at Jilin agriculture University and reproduced in our lab by feeding with wheat bran. The accumulated eggs were made into egg cards with embryo killing treatment and stored in a lab refrigerator (at 5 °C) until they were ready for use.

All tested insects were used in the experiment after three generations of reproduction in the laboratory.

### 2.2. Method

#### 2.2.1. *O. sauteri* Nymphs and Adults Preying on the Tea Thrips

In the present work, the 2nd nymph, 4th nymph, and 3-day-old *O. sauteri* and adult tea thrips (in day 3 of the adult stage) were used. The insect body color of the 2nd nymph is light yellow, and the 4th nymph is deep yellow with a longer body length [14]. The 2nd nymphs and 4th nymphs, as well as *O. sauteri* adults, were introduced to tea thrip adults in different densities. The various prey densities were, respectively, 5, 10, 15, 20, and 25 tea thrips per jar (25 mL, Zhibaozhai Insect Specimen Equipment Company, Hangzhou, China). After 24 h of predation, the predation of *O. sauteri* survival on tea thrips in each jar was counted and recorded, where the death number of thrips was determined by counting those dead bodies or deflated ones that had been sucked [15]. Six repetitions were accordingly set. A corresponding number of tea thrips was set as a blank control to observe natural death and correct the survival after predation by the *O. sauteri.* This test was carried out at 25 °C in climate-controlled incubators (Ningbo Jiangnan Instrument Factory, Ningbo, China) with 75 ± 5% RH, 16 h: 8 h L/D.

#### 2.2.2. *O. sauteri* Adults Preying on Tea Thrips Adults at Different Temperatures

A density–gradient treatment was set for one *O. sauteri* adult with 5, 10, 15, 20, and 25 tea thrips adults (3-day old adult), respectively, and six repetitions were accordingly set. In each treatment, the *O. sauteri* adult was pretreated with 24 h starvation, and then its predation on tea thrips was observed and recorded over 24 h. Additionally, the temperature conditions of 15, 20, 25, and 30 °C were accommodated in climate-controlled incubators with 75 ± 5% RH, 16 h: 8 h L/D.

#### 2.2.3. Intra-Specific Interference Response of Predators

In this test, 30 tea thrips were placed in 25 mL plastic jars. Additionally, in each insect jar, 1, 2, 3, and 4 *O. sauteri* at different insect ages were introduced to account for gradient–density. Additionally, each density test was repeated six times with three parallels. The temperature was set at 25 °C, and the RH was 85 ± 5% under a photoperiod of 16L: 8D. The number of living tea thrips was recorded over 24 h and the results were calculated.

#### 2.2.4. Response of *O. sauteri* to Population Density of Tea Thrips

In total, 100 tea thrips in the egg-laying period were housed in each 150 mL plastic jar, with gradient amounts of *O. sauteris* adults of 0, 1, 2, and 3. They were put in insect jars with a few fresh tea leaves as food and cultured in an artificial climate chamber (25 °C, 75 ± 5% RH, 16L:8D). As such, the benefit/harm ratios were set at 0:100, 1:100, 2:100, and 3:100. The group including no *O. sauteris* (benefit/harm = 0:100) was set as the control. Each treatment was repeated six times. The insects were observed every day to record the population growth and decline of the tea thrips until all of them were killed.

### 2.3. Data Analysis

#### 2.3.1. Predatory Function

The experimental data were analyzed with Holling’s disc equation and checked for their fit with any type of functional response; for example, Na = aTN/(1 + aThN) [16,17]. In this formula, “N” represents the initial density of prey; “Na” is the number of prey encountered per predator; “a” denotes the instantaneous attack rate; “T” represents the time that predator and prey are exposed to each other (1 d); and “Th” denotes the “handling time” associated with each prey eaten.

The effects of *O. sauteri* searching for the adult tea thrips can be calculated as [18]:

S = a/(1 + aTh N)

#### 2.3.2. *O. sauteri* Disturbance Response

The experimental data were simulated by Watt’s interference and competition model [19], that is, A = aX^−b^. Type “X” is the density of *O. sauteri*, type “A” is the number of tea thrips preyed, type “a” is the attack rate without competition, and type “b” represents the intra-specific competition parameter.

## 3. Results

### 3.1. Predatory Effects of O. sauteri on the Adult Tea Thrips

**Figure 1:**
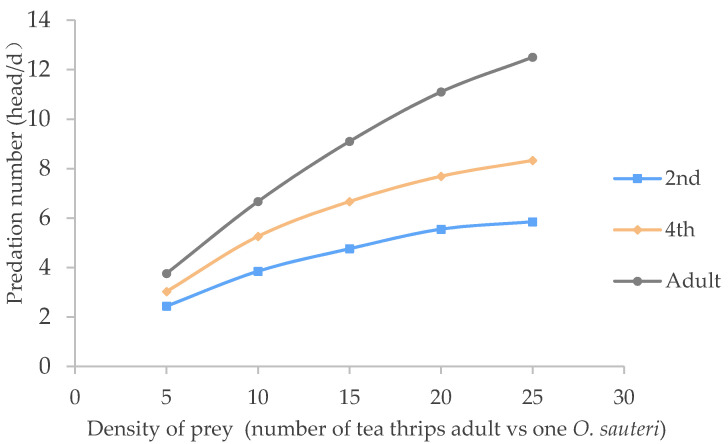
Predatory functional responses of *O. sauteri* on 2nd nymph, 4th nymph, and adult stage for adult tea thrips.

*Orius sauteri* were observed for their predatory ability at the 2nd nymph, 4th nymph, and adult stages. As previously described, the number of tea thrips per group was set as 5, 10, 15, 20 and 25. The result indicated that *O. sauteri* in each group could prey on tea thrips and was more nectivorous during the adult stage than the nymphal stage. At the same time, their predation ability was elevated when the number of tea thrips increased. Additionally, their predation ability increased with an increase in *O. sauteri*. It was obvious that the adult predator possessed a higher predation ability than both the 4th nymphs and the 2nd nymphs (Figure 1). According to the Holling type II predator–prey model [16,17], the functional responses and parameters are listed in Table 1. The predation ability of *O. sauteri* on tea thrips increased with age, and the instantaneous attack rate (a) and predatory capacity (a/Th) also increased. For the adult *O. sauteri*, the handling time was 0.0327 d, the instantaneous attack rate was 0.8573, and the upper limit of predation was 30.5 head/day. Under the same conditions, the predation ability of the *O. sauteri* on the adult tea thrips was in the order of adult > 4th nymph > 2nd nymph.

### 3.2. Temperature Affected Predation Ability of O. sauteri Adults

**Figure 2:**
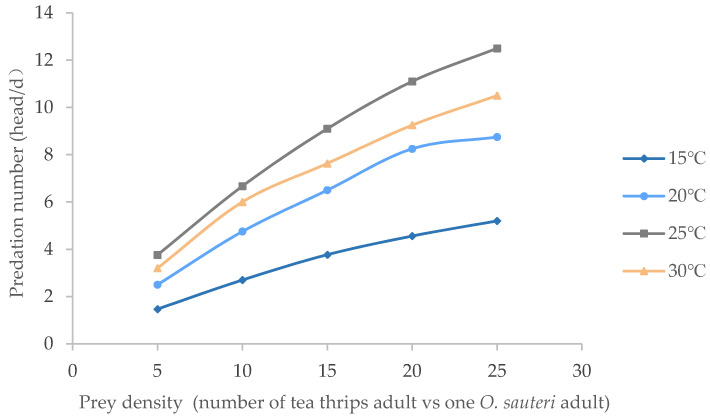
Predatory functional responses of *O. sauteri* adults on adult tea thrips at different temperatures.

The findings showed that the predation effects of *O. sauteri* adults on tea thrip adults intensified with the elevation of predation density and temperatures (Figure 2). At each given temperature, the predation number grew with increasing prey density. At the same density, the predation ability of *O. sauteri* adults increased when temperatures increased from 15 °C to 25 °C. However, when the temperature reached 30 °C, the predation number fell between the curve of 20 °C and 25 °C.

According to the Holling type II predator–prey model, the functional responses of *O. sauteri* to adult tea thrips at different temperatures were calculated (Table 2). The results showed that the correlation coefficient R was high (0.9968, 0.9814, 0.9982, 0.9833) and demonstrated that the *O. sauteri* adults at the tested temperature were significantly correlated with prey density. Additionally, the *O. sauteri* performed best at 25 °C in terms of instantaneous attack rate (0.8573), and the upper limit of predation was 30.5 per day.

### 3.3. Searching Effects of O. sauteri for the of Adult Tea Thrips

**Figure 3:**
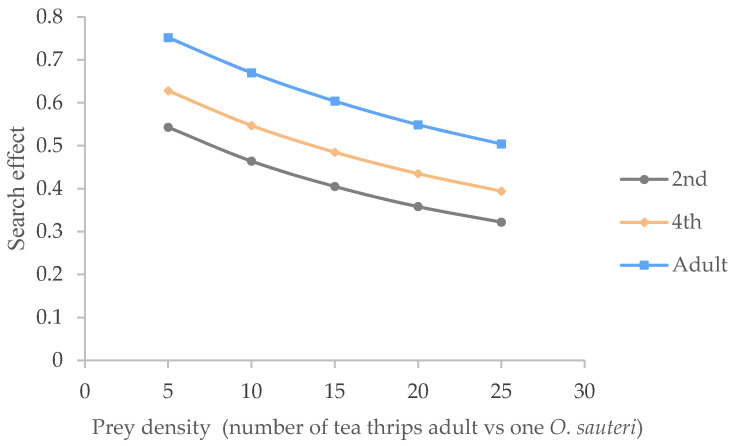
Searching effect of different stages of *O. sauteri* for adult tea thrips (25 °C).

As illustrated in Figure 3, the searching effects of the 2nd nymphs, 4th nymphs, and adults of the *O. sauteri* for adult tea thrips were investigated in relation to environmental temperature. The searching effect of *O. sauteri* at each stage gradually fell with an increase in prey density from 5 to 25. Meanwhile, at the same density of tea thrips, the searching effects of *O. sauteri* increased from the 2nd nymphs to the 4th nymphs and the adults. 

**Figure 4:**
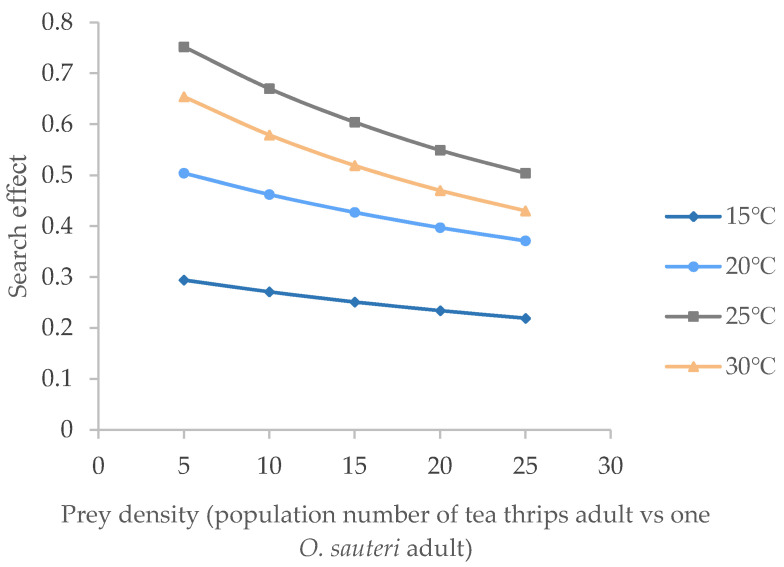
Searching effect of *O. sauteri* adults on adult tea thrips at different temperatures.

In addition, the influence of environmental temperature on this searching effect is illustrated in Figure 4. At each given temperature, the searching effect of *O. sauteri* adults declined with increasing prey concentrations; for example, the searching effect of the 2nd nymphs fell from 0.54 to 0.3, the 4th nymphs fell from 0.62 to 0.40, and the adults fell from 0.76 to 0.54. At same density of tea thrips, the searching effect increased in the order of 15 °C, 20 °C, 30 °C, 25 °C, indicating that *O. sauteri* adults had the strongest searching capability at 25 °C.

### 3.4. Intra-Specific Interference Response of O. sauteri

At different insect development periods, the intra-specific interference response of *O. sauteri* adults to its own population density is shown in Table 3. At each insect stage, with the same population of the adult tea thrips, the predation rate of *O. sauteri* decreased along with the increase in its own density. With the development of predator insect age from 2nd nymph to adult, the predation rate of *O. sauteri* at the same density increased. There was intra-specific competition and a mutual interference among *O. sauteri* adults.

### 3.5. The Effects of O. sauteri on Population Density of Tea Thrips

**Figure 5:**
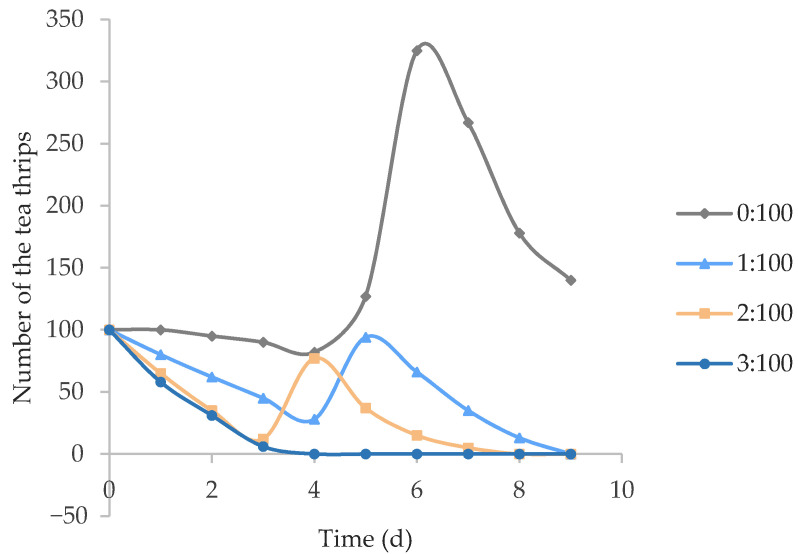
Changes in population of tea thrips under different benefit/harm ratios (number of *O. sauteri*/number of tea thrips).

The effects of *O. sauteri* on the population density of tea thrips are illustrated in Figure 5. When the benefit-to-harm ratio was 0:100 (no *O. sauteri* existing), the population density of the tea thrips was stable for the first four days, showing a slight decrease. From the fourth day, the newly spawned tea thrips began to hatch, and the population went through growth spurts and reached a peak of 325 tea thrips at the sixth day; then, the rate dropped quickly in the last three days.

When the benefit-to-harm ratio was set as 1:100, the curve basically went down over the first four days and reached its highest amount (around the same level as the initial 100 individuals) on the fifth day; it then declined to zero on the last day. When the benefit-to-harm ratio was set at 2:100, the curve ran similarly to the former one (1:100), except the highest value of tea thrips appeared on the fourth day. When the benefit-to-harm ratio was 3:100, the curve of the population density decreased from the first day and reached 0 on the fourth day, flattening continuously.

## 4. Discussion

The functional response of a predator plays a pivotal role in the population dynamics of predatory systems [20]. A few studies have reported on the functional responses of *O. sauteri* to other pests [21,22,23,24,25,26].

The present study found that the functional response of *O. sauteri* to tea thrips was characterized by a decelerating intake rate (Figure 1 and Figure 2) As the number of tea thrips increases while holding the O. sauteri constant, the number of tea thrips killed increases and then levels off. This is because the proportion of tea thrips killed per *O. sauteri* decreases as the tea thrip density increases. The higher the density of tea thrips, the smaller the proportion of caribou killed per wolf (Table 3). These functional response characters fit with Holling’s type II predator–prey model [20].

The predation ability of *O. sauteri* for adult tea thrips increased with the predator’s age in the order of adult > the 4th nymph > the 2nd nymph. The adult *O. sauteri* had the strongest predation capability for tea thrips at 25 ℃, and their predation rate was 23–62.3%. In the same temperature treatment, the higher the prey density, the larger the predation number found and the lower the finding efficiency.

In terms of potential control responses for increasing the benefit-to-harm ratio, the population density of adult tea thrips initially experienced a decline, and then recovered to its starting value and declined to zero. This might be caused by an increase in hatching tea thrips eggs, while the predation of the *O. sauteri* significantly impacted populations. When large numbers of eggs begin hatching, the population density of the tea thrips begins to grow rapidly; however, self-competition and predation by natural enemies made it drop to zero. These results are similar to those in Shang et al.’s report [27]. For tea thrips with no enemy predation, it is easy to understand that individuals create intensive intraspecific competition for food, space, light, mates, or any other resource required for survival.

## 5. Conclusions

This study preliminarily clarified the functional response and searching effects of *O. sauteri* on adult tea thrips and found that the former had biological control potential against this pest. The present results may provide a reliable basis for the proper assessment of the potential of natural enemies in controlling tea thrips. *O. sauteri* is expected to be a sustainable biocontrol method for tea garden pests.

## Figures and Tables

**Table 1:** Predatory functional responses of *O. sauteri* preying on tea thrip adults.

*O. sauteri*	Functional Response Equation	R	Handling Time (Th)/Day	Instantaneous Attack Rate (a)	Predatory Capacity (a/Th)	Predation Upper Limit (No./Day)
2nd nymph	1/N_a_ = 1.5262/N + 0.1051	0.9429	0.1051	0.6552	6.420	9.500
4th nymph	1/N_a_ = 1.356/N + 0.0588	0.9757	0.05882	0.7373	12.53	17.00
Adult	1/N_a_ = 1.1664/N + 0.0327	0.9982	0.03270	0.8573	26.21	30.50

**Table 2:** Predatory functional responses *O. sauteri* adults preying on adult tea thrips at different temperatures.

Temp. (°C)	Functional Response Equation	R	T_h_ (d)	a	a/T_h_	Predation Upper Limit (No./d)
15	1/N_a_ = 3.0948/N + 0.059	0.9968	0.0590	0.3231	5.480	17.00
20	1/N_a_ = 1.809/N + 0.0354	0.9814	0.03540	0.5528	15.61	28.00
25	1/N_a_ = 1.1664/N + 0.0327	0.9982	0.03210	0.8573	26.21	30.50
30	1/N_a_ = 1.3603/N + 0.04	0.9833	0.04000	0.7531	18.82	25.00

**Table 3:** Predation rate on tea thrips and intra-specific interference response.

Insect Stage	No. of *O. sauteri VS* 30 Tea Thrips (X)	No. of Total Predation (A)	Predation Rate per *O. sauteri*	Intra-Specific Interference Response Equation	R^2^
2nd nymph	1	7.3	24.3%	A = 7.9026X^−0.933^	0.9335
	2	9	15%		
	3	9.9	11%		
	4	7.2	6%		
4th nymph	1	15.3	51%	A = 15.953X^−0.629^	0.9783
	2	22.4	37.35		
	3	24	26.7%		
	4	25.6	21.3%		
Adult	1	18.7	62.3%	A = 19.257X^−0.523^	0.9887
	2	25	41.7%		
	3	25.8	28.7%		
	4	27.6	23%		

## Data Availability

No other data supporting report available.

## References

[1] Singh H.R., Hazarika P. (2020). Biotechnological approaches for tea improvement. Biotechnological Progress and Beverage Consumption.

[2] Jones D.R. (2005). Plant viruses transmitted by thrips. Eur. J. Plant Pathol..

[3] Settele J., Biesmeijer J., Bommarco R. (2008). Switch to ecological engineering would aid independence. Nature.

[4] Gurr G.M., Wratten S.D., Snyder W.E., Read D.M.Y. (2012). Biodiversity and Insect Pests: Key Issues for Sustainable Management.

[5] Chailleux A., Mohl E.K., Alves M.T., Messelink G.J., Desneux N. (2014). Natural enemy-mediated indirect interactions among prey species: Potential for enhancing biocontrol services in agroecosystems. Pest Manag. Sci..

[6] Li H.L., Li P., Zhang H., Wang D.F., Li L.D., Zeng M.S., Wu G.Y., Wang Q.S. (2019). Predation of *Orius sauterion* pest insects of pea pushes. Acta Teas Sin..

[7] Nagai K., Yano E. (2000). Predation by *Orius sauteri* (Poppius) (Heteroptera: Anthocoridae) on *Thrips palmi* Karny (Thysanoptera: Thripidae). Functional response and selective predation. Appl. Èntomol. Zool..

[8] Yin J., Gao X.G., Wu Y.Q. (2013). Thrips control on the greenhouse eggplant by releasing *Orius sauteri* (Heteroptera: Anthocoridae). Chin. J. Biol. Control.

[9] Guo J.Y., Wan F.H. (2001). Use Kalanchoe bolssfeldiana as oviposition plant for mass rearing *Orius sauteri* (Hemiptera: Anthocoridae). Chin. J. Biol. Control.

[10] Zhang W. (1980). A brief description of the species and biology of common *Orius insidiosus* (Hemiptera: Anthocoridae). Nat. Enemies Insects.

[11] Wenjun B., Leyi Z. (2011). Chinese Animal Records: Insects.

[12] Landis D.A., Wratten S.D., Gurr G.M. (2000). Habitat management to conserve natural enemies of arthropod pests in agriculture. Annul. Rev. Entomol..

[13] Zhang R., Ji D., Zhang Q., Jin L. (2021). Evaluation of eleven plant species as potential banker plants to support predatory *Orius sauteri* in tea plant systems. Insects.

[14] Ha Q.H. (2002). Study on morphological, biological, ecological characteristics of predacious bug *Orius Poppius* (Hemiptera: Anthocoridae) reared on *Thrips palmi* Karny and eggs of *Corcyra cephalonica* Staintou. Proc. Vietnam. Natl. Conf. Entomol..

[15] Nagai K., Yano E. (1999). Effects of temperature on the development and reproduction of *Orius sauteri* (Poppius) (Heteroptera: Anthocoridae), a predator of *Thrips palmi* Karny (Thysanoptera: Thripidae). Appl. Entomol. Zool..

[16] Holling C.S. (1959). Some characteristics of simple types of predation and parasitism. Can. Ent..

[17] Wu K.J., Sheng C.F., Gong P.Y. (2004). Equation of predator functional response and estimation of the parameters in it. Entomol. Knowl..

[18] Ding Y.Q. (1994). Mathematical Ecology of Insects.

[19] Hassell M.P., Verley G.C. (1969). New inductive population model for insect parasite and its bearing on biological control. Nature.

[20] Schenk D., Bacher S. (2010). Functional response of a generalist insect predator to one of its prey species in the field. J. Anim. Ecol..

[21] Bing L., Meng S., Yifan Z., Hao C., Yi Y., Li Z. (2017). Evaluation of the biocontrol capacity of predatory bug *Orius sauteri*, reared on *Sitotroga cerealella* eggs, on *Thrips palmi* based on predatory functional response. J. Plant Prot..

[22] Bing L.V., Sun M., Zhai Y.F., Chen H., Yi Y.U., Zheng L. (2018). Effection of short adaptive pre-feeding on the predatory functional response to *Orius sauteri* reared on *Sitotroga cerealella* eggs. J. Environ. Entomol..

[23] Fu B.L., Qiu H.Y., Li Q., Sun Y.T., Zhou S.H., Yang S.Y., Shan-guang L., Tang L.D., Zhang F.P., Liu K. (2019). Predation of *Orius sauteri* on Thrips hawaiiensis in the laboratory. Chin. J. Appl. Entomol..

[24] Han L.L., Dong T.Y., Zhao K.J., Zhu M.H., Sun W.P., Xu Z.X., Shi L. (2015). Predation of Aphis glycines by *Orius sauteri* nymphs. China J. Biol. Control..

[25] Liang Z., Ge Z.T., Gong Y.J., Shi B.C., Su W., Wei S.J. (2015). Effects of temperature on predation of the thrips *Echinothrips americanus* (Thysanoptera: Thripidae) by the predatory bug *Orius sauteri* (Heteroptera: Anthocoridae). J. Plant Prot..

[26] Zhi J.R. (2011). The predation of Orius similis to Frankliniella occidentalis and Aphis craccivora. Chin. J. Appl. Entomol..

[27] Shang S.Q., Liu P., Chen Y.N., Zhang X.H. (2017). Functional response and control potential of *Neoseiulus barkeri* to *Tetranychus urticae*. Plant Prot..

